# Bibliometrics analysis on the research status and trends of small bowel adenocarcinoma: 1923-2023

**DOI:** 10.3389/fonc.2024.1407315

**Published:** 2024-08-21

**Authors:** Yao Lu, Cheng C. Pan, Xin Hu, Jing Sun

**Affiliations:** ^1^ Department of Community Nursing, School of Nursing, Peking University, Beijing, China; ^2^ Nanjing University of Finance and Economics, Nanjing, China

**Keywords:** small bowel adenocarcinoma[MeSH], Citespace, Web of Science Core Collection, bibliometrics [MeSH], trends, epidemiology

## Abstract

**Objectives:**

The aim of this research is to discuss the research status, hotspots, frontiers, and development trends in the field of small bowel adenocarcinoma based on bibliometrics and visual analysis by CiteSpace software.

**Methods:**

The relevant research articles on SBA from 1923 to 2023 were retrieved from the Web of Science Core Collection database. CiteSpace software was used to form a visual knowledge map and conduct analysis for the countries/regions, journals, authors, keywords, clusters, research hotspots and frontiers of the included articles.

**Results:**

There were 921 articles included, and the number of articles published during 1923-2023 is increasing. The country with the highest number of articles published was the United States (443, 38.76%), followed by Japan (84, 9.12%) and France (72, 7.82%). The author with the highest number of publications is Ansell, Overman MJ (33, 3.58%), and the author with the highest co-citation frequency is Overman MJ (218). Journal of Clinical Oncology is the journal with the highest publication frequency. The top five cluster groups were “chemotherapy”, “inflammatory bowel disease”, “celiac disease”, “tumor” and “small intestine”. The related disease, chemotherapy drugs, and treatment regimens of SBA form the main research fields, and prognosis and diagnosis are the research hotspots and trends.

**Conclusion:**

The global research field in SBA has expanded in the past 100 years. The prognosis and new diagnosis of SBA are hotspots in this field and require further study in the future.

## 1 Introduction

Small bowel adenocarcinoma (1, 2) is a rare malignancy of the gastrointestinal tract (3). According to EUROCARE data, the annual number of new cases of SBA estimated in Europe is 3600 (4). The incidence of SBA increased from 1.18 per 100,000 in 1973 to 2.27 per 100,000 in 2004 in the United States (5). The incidence rate of SBA in France increased from 0.69 to 0.8 per 100,000 in men, and from 0.37 to 0.51 per 100,000 in women between 1996-2000 and 2011-2015 (1). In the Netherlands, the age-standardized incidence of SBA increased from 0.5 per 100,000 in 1999 to 0.7 per 100,000 in 2013 (6). SBA is mostly diagnosed in the late stage, the median age at diagnosis is around 60 years, of which over 85% present above 50 years of age and with a relatively higher incidence among males (relative risk: 2.6 for males, 2 for female) (7). SBA, especially in the duodenum, has a poorer prognosis compared with colorectal cancer, with overall 5-year survival as low as 26 to 41%, and median overall survival (OS) of 20 to 38 months (8). Patients with metastatic SBA have an overall 5-year survival of 11 to 19%, which is similar to metastatic colorectal cancer (9).

The symptoms for SBA are non-specific, leading to delayed diagnosis in many patients (10). In a study of 217 patients with SBA, most of the patients (66%) had abdominal pain at the time of diagnosis, and emergency diagnosis with occlusion or bleeding was reported in 40% and 24%, respectively (3). About 60% of patients are symptomatic at presentation, and the most common symptom is related to stenosis (11). Besides, the symptomatic presentation of SBA was more common for jejuno-ileal primaries (84%) as compared to duodenum (54%) (12).

At present, the pathogenesis is still controversial (13). Most SBA arise from malignant transformation of small bowel adenomas, familial intestinal polyps, Peutz-Peutz disease, and Crohn’s disease (14). At the molecular level, Genomic profiling studies have identified a number of critical molecular drivers in the pathogenesis of SBA, including E-cadherin, KRAS, TP53, and SMAD (14). SBA has also been associated with a higher likelihood of microsatellite instability and high tumor mutational burden (15). At the macro level, the increased risk for SBA appears to correlate between the severity of duodenal polyposis and presence of jejunal polyps (16). A systematic review of associations between lifestyle risk factors and SBA suggests that smoking and intakes of red/processed meat, alcohol and sugary drinks are associated with a higher risk of SBA (16). One study of associations between occupation risk factors and SBA suggested that building caretakers, housekeepers, general farm laborers, dockers, dry cleaners, textile workers and welders were at risk for SBA (17).

SBA is always diagnosed at an advanced stage due to atypical symptoms and limited sensitivity to conventional radiological imaging (18). According to reports from studies in recent 20 years, most diagnoses in patient samples were made by endoscopy and surgery (19). So far, no specific cancer markers have been described for SBA (20). However, elevated levels of CEA (20–50%), CA 19-9 (29.2–44.4%) may have a prognostic value, especially in the advanced disease stages (21). Before 2000, the median time from initial medical evaluation to diagnosis was about 6-8 months (22, 23); after 2000, this time has significantly shortened to about 30 days (24).

As recommended in clinical practice guidelines, all the regimens of SBA were similar to those used in the treatment of colorectal cancer (25). However, recent data on molecular profiling have highlighted the settings where it may not be possible to treat SBA as an extension of CRC (13). In Japan, the effectiveness and safety of the folinic acid, fluorouracil, and oxaliplatin regimen for SBA have been reported (26). As described in the National Comprehensive Cancer Network guideline (27), the combination of fluoropyrimidine with irinotecan (such as folinic acid, fluorouracil, and irinotecan) may be an alternative option (28). The only method of radical treatment of SBA is surgical resection and its extension depends on the location of a primary lesion and disease stage (29). So far, the prognosis of SBA remains poor, with a 5-year overall survival (OS) of ~55% and ~4%, for stage I and stage IV disease, respectively (30).

Few reports have analyzed the characteristics and development trend of the SBA over a long period of time, which is not conducive for researchers to accurately grasp the occurrence, development rules and characteristics of the SBA (31). With an increasing number of reports on the SBA research, retrieving the research status quickly and efficiently in related fields has become a more realistic problem faced by researchers (32). Bibliometrics and visual analysis provide an important, feasible and systematic method for judging the importance of published literature by showing the author’s networks and academic exchanges, connections between scholars and the development in the field of knowledge (33). Using the results of the bibliometric analysis will not only help researchers understand the global research trends of SBA and master the information sources of SBA research but also help researchers understand the advantages and disadvantages of their research and quickly capture the research priorities, hotspots, and trends (34).

In this study, the research articles related to the small bowel adenocarcinoma in Web of Science Core Collection database were selected and analyzed by using CiteSpace software. From the perspectives of bibliometrics and visual analysis, the research progress of SBA is discussed, aiming to understand the research development trends and new trends of SBA, identify the hotspots in this research field, and provide a reference and basis for better research on SBA.

## 2 Materials and methods

### 2.1 Data source and search strategy

Web of Science Core Collection (WOSCC) is a comprehensive and multidisciplinary citation index database(http://www.webofscience.com). It was established by Clarivate Analytics and has been widely used by researchers and academics since its introduction. The database covers a vast array of scholarly journals from various disciplines, including science, social sciences, humanities, and engineering (35). One of the distinctive features of WOSCC is the inclusion of citation information. It enables users to track the impact and influence of specific articles and authors through citation analysis. This feature is crucial for researchers who want to evaluate the significance of a specific study, identify key contributors in a field, or track the development of a research topic over time (36).

We retrieved the Web of Science Core Collection database, and the search formula is as follows: TS=(“adenocarcinoma of the small bowel” OR “adenocarcinoma of small gut” OR “adenocarcinoma of small intestin*” OR “small intestin* adenocarcinoma” OR “small bowel adenocarcinoma” OR “glandular cancer of the small bowel” OR “glandular cancer of small gut” OR “glandular cancer of small intestin*” OR “small intestin* glandular cancer” OR “small bowel glandular cancer”). The search time range was from 1923 to 2023. The last retrieval date was December 31, 2023.

### 2.2 Inclusion and exclusion criteria

The periodical articles with research contents related to the theme of “small bowel adenocarcinoma” were included by reading the titles, abstracts and keywords of the detected articles. All types of articles were included. Articles with duplicate articles were excluded.

### 2.3 Analyzing tools and statistical methods

CiteSpace is a tool that utilizes co-citation networks to analyze and visualize research (37). It predicts the future development of a field by analyzing information from articles (38). Using the CiteSpace software, a visual co-occurrence network was generated, with parameters set to a time span from January 1923 to December 2023 and a time slice of one year. The top 10 high-frequency nodes are selected per time slice using the “Top N” threshold item, with “Pathfinder” as the selected connection mode to simplify the network structure and emphasize important features.

## 3 Analysis results and visualization

### 3.1 Published outcomes and cited outcomes

**Figure 1:**
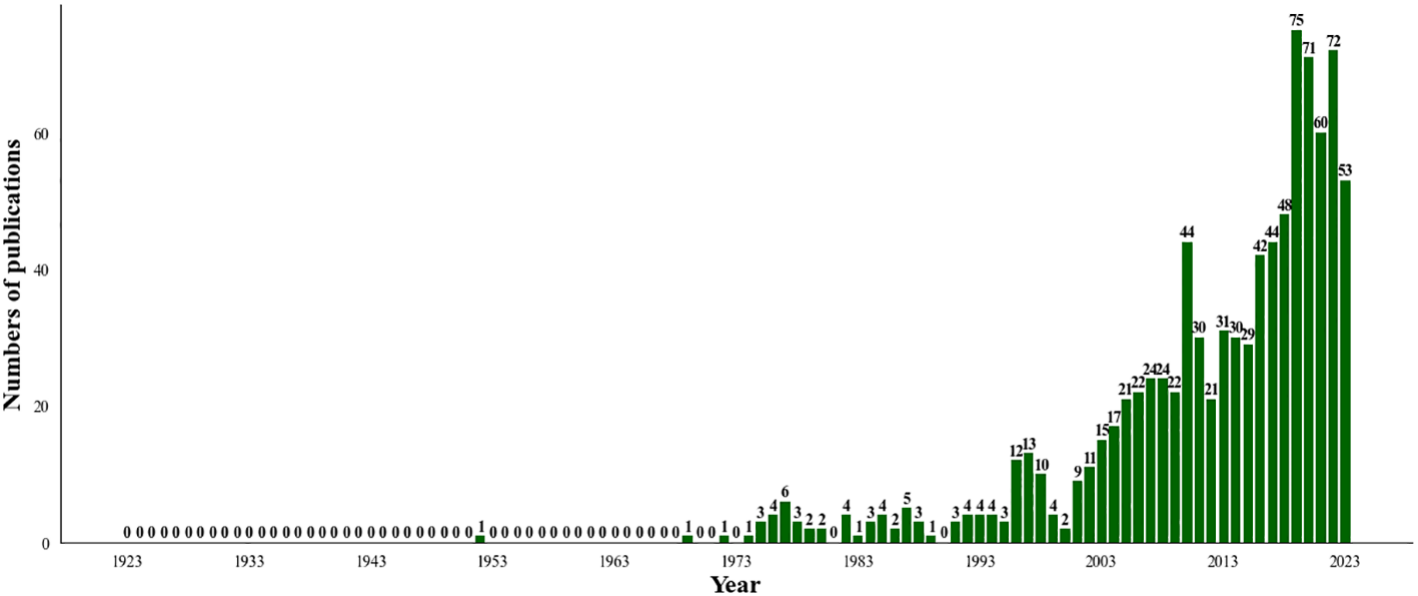
The annual quantities of SBA articles from 1923 to 2023.

A total of 923 articles were retrieved, and duplicated articles in the imported articles were deleted by using CiteSpace software. 921 articles were included. The number of articles published in the past decade has shown a steady growth trend. In 2022 (72), the number of publications was approximately 2.3 times that in 2013 (31). See **Figure 1** for details.

The top5 research areas of SBA are Oncology(348, 37.785%), Gastroenterology Hepatology(270, 29.316%), Surgery(139, 15.092%), Pathology(92, 9.989%), General Internal Medicine(82, 8.903%). See **Table 1** for details.

**Table 1:** The research areas of SBA articles from 1923 to 2023.

Rank	Areas	Publications	%(N=921)	Rank	Areas	Publications	%(N=921)
1	Oncology	348	37.785	11	Cell Biology	10	1.086
2	Gastroenterology Hepatology	270	29.316	12	Life Sciences Biomedicine Other Topics	10	1.086
3	Surgery	139	15.092	13	Science Technology Other Topics	10	1.086
4	Pathology	92	9.989	14	Biochemistry Molecular Biology	8	0.869
5	General Internal Medicine	82	8.903	15	Microbiology	8	0.7
6	Radiology Nuclear Medicine Medical Imaging	32	3.474	16	Psychology	8	0.7
7	Research Experimental Medicine	25	2.714	17	Transplantation	8	0.7
8	Veterinary Sciences	19	2.063	18	Biophysics	7	0.76
9	Pharmacology Pharmacy	13	1.412	19	Agriculture	7	0.612
10	Public Environmental Occupational Health	11	1.194	20	Immunology	6	0.651

Among the 921 retrieved articles, the total citation frequency was 14615, the average citation frequency of each article was 15.87 times. *Munkholm et al’s study* in *Alimentary Pharmacology & Therapeutic* in 2003 has the highest citation frequency of 414 times.

The top 10 most frequently cited articles are shown in **Table 2**.

**Table 2:** Top 10 highly cited articles from 1923 to 2023.

Rank	Tiltle	Total citations	Publication year	Journal	Impact factors
1	Review article: the incidence and prevalence of colorectal cancer in inflammatory bowel disease	414	2003	ALIMENTARY PHARMACOLOGY & THERAPEUTICS	7.60
2	Celiac disease: a comprehensive current review	396	2019	BMC MEDICINE	9.30
3	Adenocarcinoma of the small bowel: Presentation, prognostic factors, and outcome of 217 patients	348	2004	CANCER	
4	Colorectal Cancer and Inflammatory Bowel Disease: Epidemiology, Risk Factors, Mechanisms of Carcinogenesis and Prevention Strategies	311	2009	ANTICANCER RESEARCH	6.20
5	Risk of malignancy in patients with celiac disease	264	2003	AMERICAN JOURNAL OF MEDICINE	2.00
6	Adenocarcinoma of the small bowel - Review of the National Cancer Data Base, 1985-1995	238	1999	CANCER	5.90
7	Cancer in inflammatory bowel disease	235	2008	WORLD JOURNAL OF GASTROENTEROLOGY	4.30
8	Small bowel adenocarcinoma: Epidemiology, risk factors, diagnosis and treatment	189	2014	DIGESTIVE AND LIVER DISEASE	4.50
9	Intestinal and extra-intestinal cancer in Crohn’s disease: follow-up of a population-based cohort in Copenhagen County, Denmark	183	2004	ALIMENTARY PHARMACOLOGY & THERAPEUTICS	7.60
10	Phase II Study of Capecitabine and Oxaliplatin for Advanced Adenocarcinoma of the Small Bowel and Ampulla of Vater	159	2009	JOURNAL OF CLINICAL ONCOLOGY	45.30

### 3.2 Journals, authors and countries/regions distribution

These articles are published in 498 journals, with an average published volume of 1.85 articles. The journal with the most published articles is Journal Of Clinical Oncology, with 43 articles, accounting for 4.669% of the total. The journals with the top 5 articles included 173 articles, accounting for 17.414% of the total, as shown in **Table 3**.

**Table 3:** Top 5 journals with the largest number of articles from 1923 to 2023.

Rank	Journal	Publications	%(N=921)	Impact factors
1	JOURNAL OF CLINICAL ONCOLOGY	43	4.669	45.3008
2	AMERICAN JOURNAL OF GASTROENTEROLOGY	41	4.452	9.8003
3	MODERN PATHOLOGY	38	3.325	7.4996
4	LABORATORY INVESTIGATION	27	2.362	5.5002
5	ANNALS OF ONCOLOGY	24	2.606	50.5012

**Figure 2:**
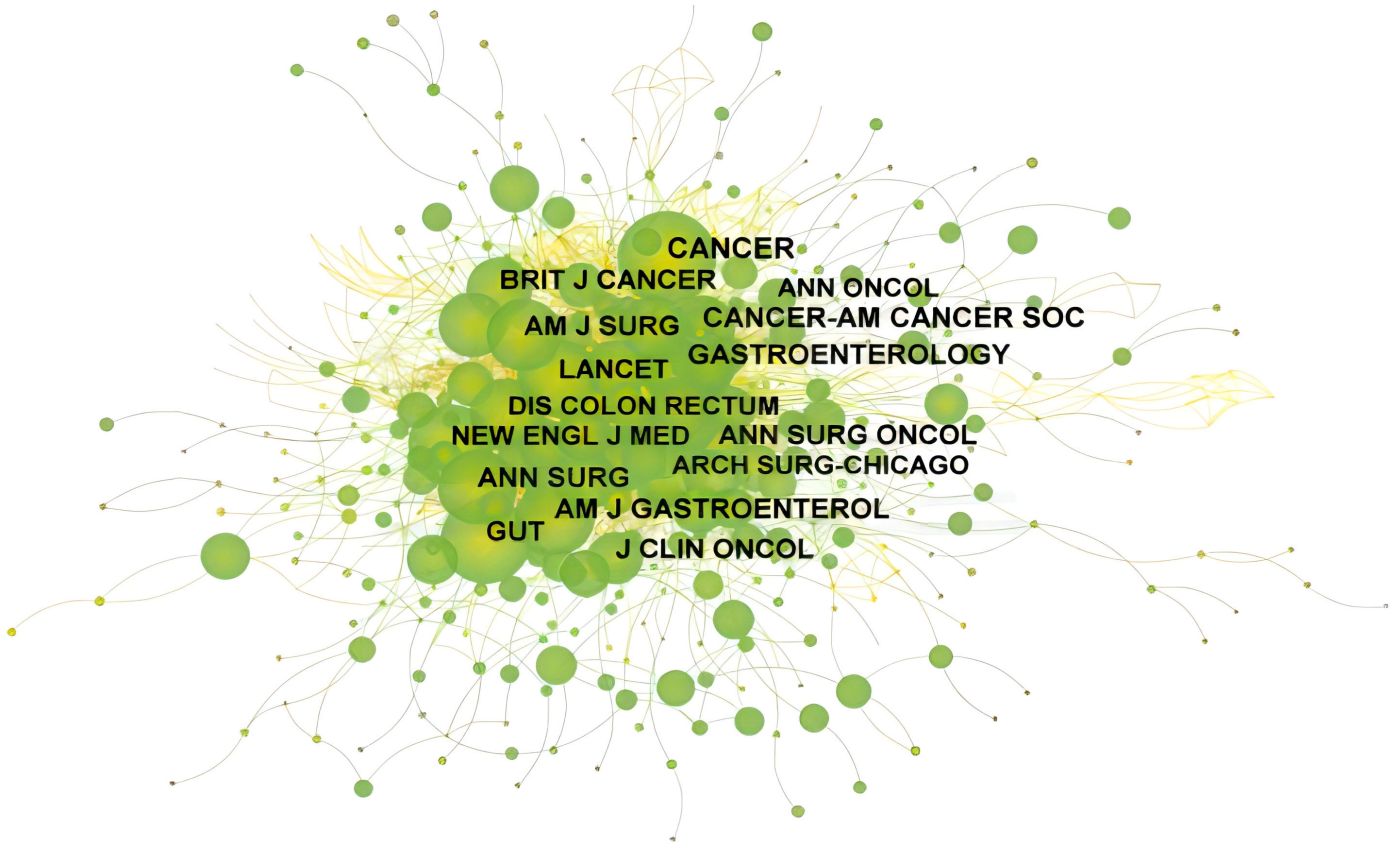
The co-citation network of SBA articles from 1923 to 2023. From:Citespace.

**Figure 2** is the co-citation network of journals, in which the number of nodes is 655 and the number of links is 2667. The top 5 cited journals are Cancer (361, 13.54%), Cancer-AM Cancer Soc (315, 11.81%), Gastroenterology (297, 11.41%), AM J Gastroenterology(267, 10.01%), and ANN Surg (260, 9.75%). Centrality reflects the importance of nodes, which is shown as a purple circle in the figure. The higher the centrality is, the more important the node is. The top 5 journals in centrality are AM J Clin Pathol (0.17), Cancer Res (0.16), AM J Clin Pathol (0.15), AM J Surg (0.14), Cancer Lett (0.14).

A total of 10498 authors are involved in the publication of articles related to SBA. Five authors write more than 20 articles, and among them, Overman MJ from UTMD Anderson Cancer Center ranks first with 33 articles. See **Table 4** for details.

**Table 4:** Top 10 authors by number of published works from 1923 to 2023.

Rank	Author	Publications	Institution
1	Overman MJ	33	UTMD Anderson Cancer Center
2	Aparicio T	28	Hospital Universitaire Saint-Louis
3	Hong SM	27	University of Ulsan College of Medicine
4	Zaanan A	25	Hospital Universitaire Europeen Georges-Pompidou
5	Stevens RH	24	Temple University
6	Svrcek M	19	Sorbonne Universite
7	Jun SY	18	Catholic University of Korea
8	Wolff Ra	18	UTMD Anderson Cancer Center
9	Afchain P	17	Hospital Universitaire Saint-Antoine
10	Osborne J W	16	Washington University in St Louis School of Medicine

**Figure 3:**
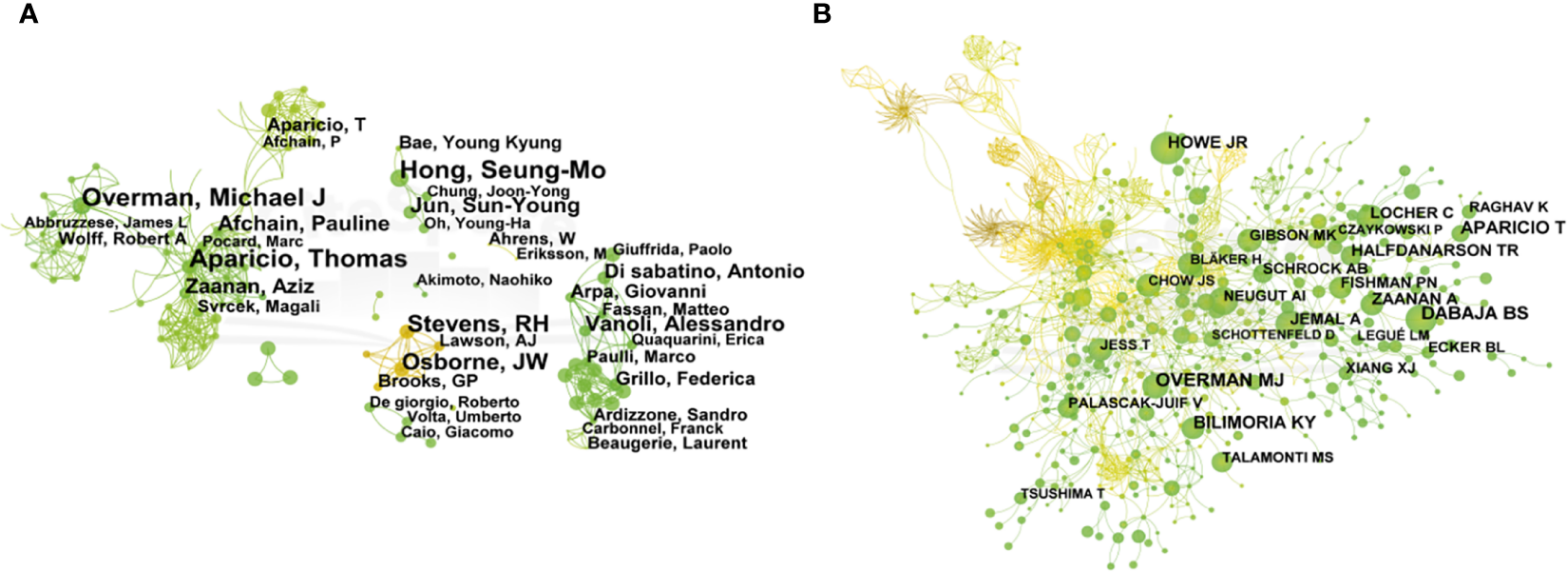
The network map of cooperation between authors **(A)** and co-authorship **(B)** of SBA articles from 1923 to 2023. From:Citespace.

In the network map of cooperation between authors, the number of nodes is 986, and the number of links is 1972, in which one node represents an author and the size of the circle represents the number of published articles by the author. The larger the node diameter is, the more published articles there are. The connection between the nodes indicates that the authors have a cooperative relationship, as shown in **Figure 3A**.

The results show that Aparicio T, Overman MJ, Hong SM, Stevens RH, OsborneJW, Afchain P, Jun SY, Zaanan A and other authors with high publication volumes each formed several independent core author groups, with close cooperation within the core author group and relatively few connections between different author groups. These authors’ research topics reflect the hot topics in the field to a certain extent, so paying attention to the research direction and content of these core author groups can better understand the development frontier and trend of SBA’s disease research.

The number of nodes in the co-authorship network is 976, and the number of links is 2800. The top 5 co-authorship times ranking are Overman MJ (218), Dabaja BS (196), Aparicio T (176), Bilimoria KY(152) and Howe JR (127), as shown in **Figure 3B**.

There are 63 countries/regions around the world paying attention to the research on SBA. Five countries have published more than 50 articles, and the top 10 countries/regions have 915 articles, accounting for 89.42% of the total published articles. Among them, USA ranks first with 443 articles, accounting for 38.76% of the total number of articles, followed by the Japan (84, 9.12%). See **Table 5** for details.

**Table 5:** Top 10 countries/regions with the largest number of articles from 1923 to 2023.

Rank	Country	Publications	%(N=921)
1	USA	443	38.758
2	JAPAN	84	9.121
3	FRANCE	72	7.818
4	PEOPLES R CHINA	66	7.166
5	ITALY	65	7.058
6	GERMANY	46	4.995
7	SOUTH KOREA	42	4.56
8	ENGLAND	41	4.452
9	CANADA	28	3.04
10	UK	28	2.45

**Figure 4:**
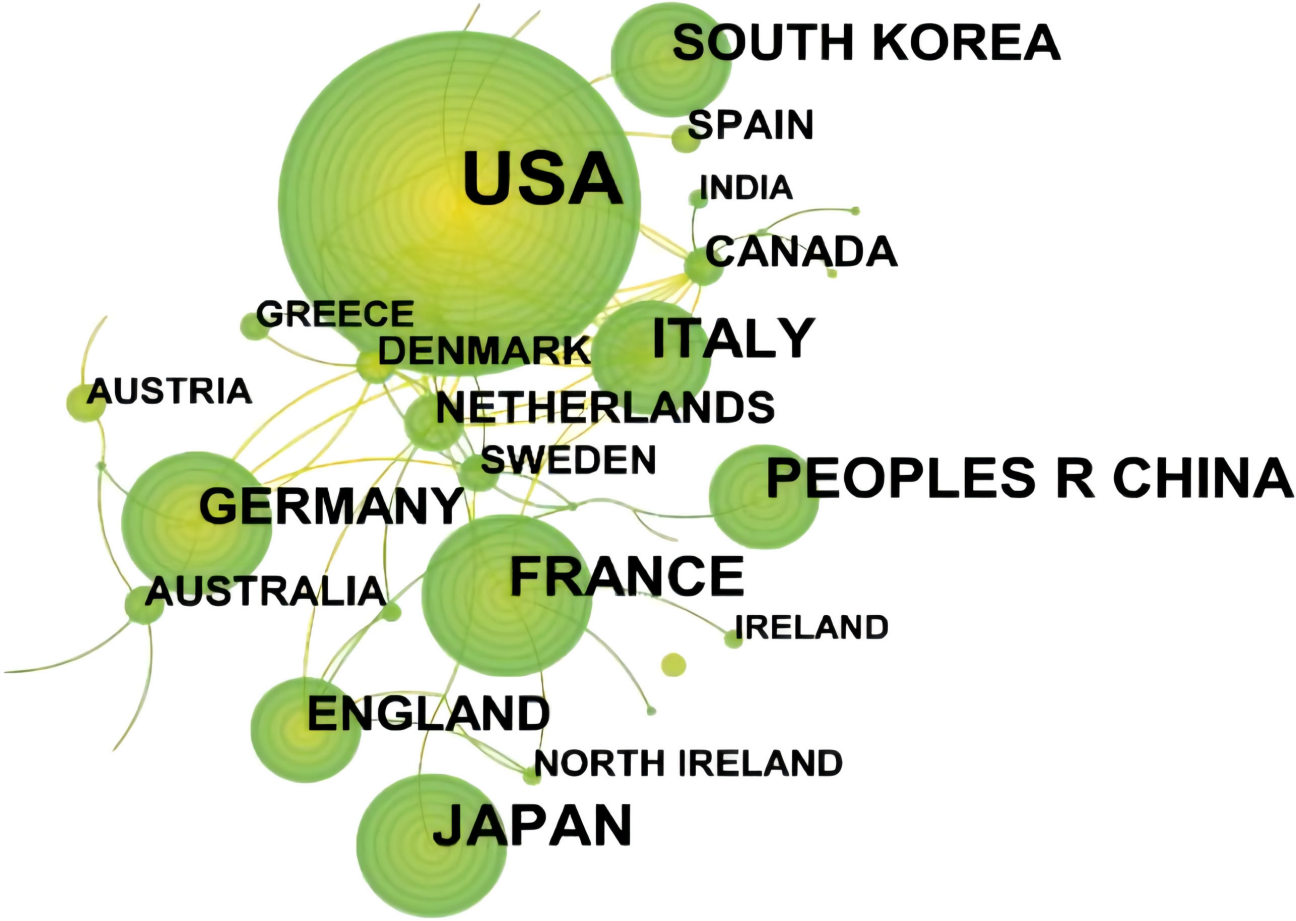
Network of cooperation among countries/regions of SBA articles from 1923 to 2023. From:Citespace.

As shown in **Figure 4**, the number of nodes in the network of cooperation among countries/regions is 55, and the number of links is 80. The top 5 countries in the centrality ranking are the Germany (0.34), Italy (0.32) and Canada (0.26), USA(0.22), Sweden(0.20), indicating that these 3 countries have close cooperation with other countries/regions.

### 3.3 Frequency, co-occurrence analysis, cluster analysis and burst detection of keywords

**Figure 5:**
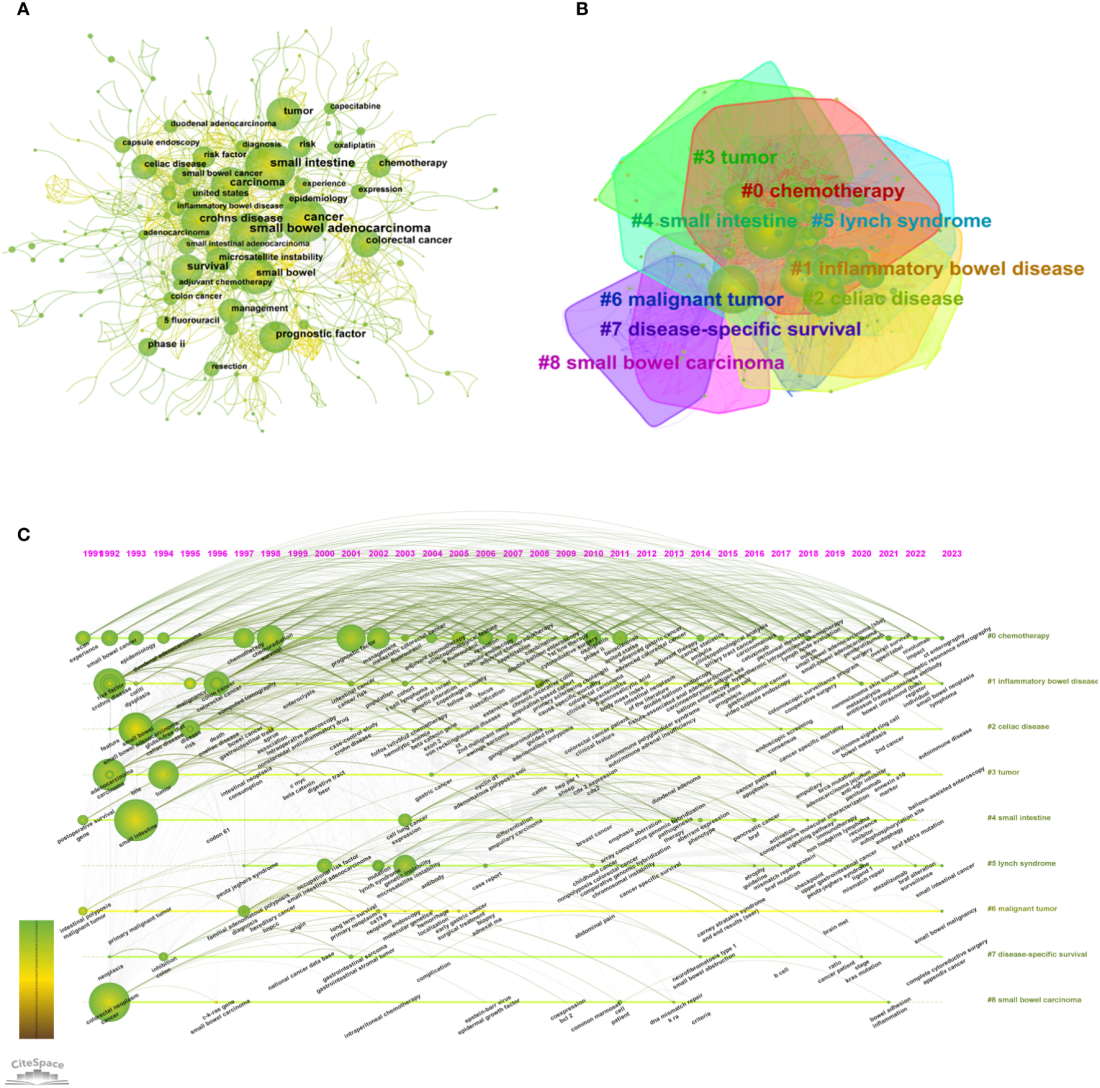
**(A)** Network of co-occurrence among keywords of SBA articles from 1923 to 2023. **(B)** Keyword clusters analysis of SBA articles from 1923 to 2023 **(C)** The timeline view of keyword clusters of SBA articles from 1923 to 2023. From:Citespace.

Using CiteSpace software to visually analyze the keywords, we obtain the network of co-occurrence among keywords, as shown in **Figure 5A**. The number of nodes in the keyword co-occurrence network is 626, and the number of links is 1,548. As shown in **Table 6**, the high frequency keywords in the top 5 are “small bowel adenocarcinoma” (278), “cancer” (188), “small intestine” (168), “Crohn’s disease” (116) and “prognostic factor” (106).

**Table 6:** High frequency keywords (≥75) of SBA articles from 1923 to 2023.

Rank	Keyword	Frequency	Centrality
1	small bowel adenocarcinoma	278	0.01
2	cancer	188	0.16
3	small intestine	168	0.02
4	crohns disease	116	0.26
5	prognostic factor	106	0.01
6	survival	102	0.00
7	carcinoma	92	0.15
8	colorectal cancer	88	0.05
9	small bowel	77	0.01
10	tumor	75	0.06

Using the log-likelihood ratio (LLR) method in keyword clustering, a total of 28 clustering groups are obtained. Each module represents a cluster, and the larger the module is, the greater the number of keywords in the cluster. The first five cluster groups are as follows: #0 chemotherapy, #1 inflammatory bowel disease, #2 celiac disease, #3 tumor, and #4 small intestine, as shown in **Figure 5B**. The clustering groups reflect that the research hotspots are mainly focused on the lesion location, differential diagnosis and therapeutic medication. The timeline view of keyword clusters mainly reflects the relationship between different clustering groups and the changing trend of keywords in the research process. A horizontal line represents a clustering group, and different keywords are arranged on the horizontal line in chronological order. Timeline View in CiteSpace was selected to visually analyze the keywords, and the results are shown in **Figure 5C**.

**Figure 6:**
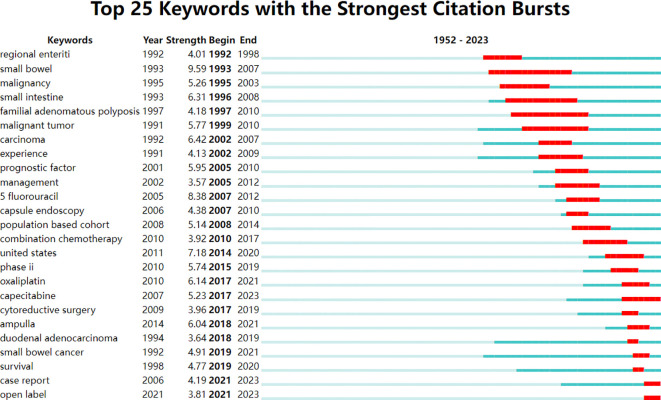
Top 25 keywords with the strongest citation bursts of SBA articles from 1923 to 2023.

Burstness refers to keywords with a sudden or significant increase in frequency in a short time (39). The larger the burst strength is, the more active the field is, and the better it can focus on research hotpots. Using the keyword burst detection function of CiteSpace software, a total of 56 burst keywords are detected, of which the top 25 are shown in **Figure 6**. Among them, “small bowel” (9.59) has the strongest burst strength. The keywords with strong burst strength in the past 10 years are “United States” (2014-2020), “phase II” (2015-2019), “cytoreductive surgery” (2017-2019), “capecitabine” (2017-2023), “oxaliplatin” (2017-2023), “duodenal adenocarcinoma” (2018-2019), “ampulla” (2018-2021), “survival” (2019-2020), “small bowel cancer” (2019-2021), “case report” (2021-2023), “open label” (2021-2023). The change in that keyword with time can be roughly divided into three stages, in which the keywords of each stage are shown in **Table 7**.

**Table 7:** Keywords during different periods.

Time Span	Keywords
1992-2007	small bowel, small intestine, ampulla, malignant tumor, malignancy, familial adenomatous polyposis, regional enteritis
2007-2015	5 fluorourcil, carcinoma, prognostic factor, capecitabine, combination chemotherapy, management
2015-2023	United States, phase ii, survival, oxaliplatin, capsule endoscopy, cytoreductive surgery

## 4 Discussion

### 4.1 Historical growth and stages of SBA research

In this study, 921 articles related to SBA from the Web of Science Core Collection were analyzed by bibliometric methods. From 1923 to 2023, the number of published articles showed an overall upward trend (**Figure 1**), which can be divided into three stages.

Before 2001, it was in the initial research period of SBA, with a few articles published and a slow growth rate of literature. The basic research in this period laid a solid theoretical foundation for the development of SBA.

Between 2001 and 2019, the number of articles published has increased rapidly, and the number of articles reached its peak by 2019. It’s probably because medical technology and research methods have improved and developed during this period. Firstly, imaging techniques have improved for diagnosing SBA (12). Especially among patients with anemia of unknown origin, the cases of SBA diagnosed as a bleeding source are increasing (39). Secondly, comprehensive molecular analysis has improved for new drug discovery and immunotherapy (12), which has enabled researchers to better understand and study.

Since 2019, there was an insignificant increased trend. Perhaps because previous research has provided a relatively complete foundation for SBA, researchers have made certain breakthroughs and progress, so the space and demand for increasing the number of studies may be relatively small.

However, slightly different from the findings of Li et al. ‘s (40) analysis shows that the trend of increasing SBA publications between 2000 and 2022 is not significant, suggesting that one of the main reasons for this is the low incidence of SBA. This difference may be mainly due to the fact that the literature data included in our study has a longer time span and is therefore more consistent with the trend of change over time.

### 4.2 Geographical distribution and international collaboration

For countries, the United States, Japan and France publish the largest number of articles (**Table 5**), and the Germany, Italy and Canada engage in relatively more international cooperation (**Figure 4**), which is basically consistent with the results of other studies (41). Firstly, this may be because rising GDP and rapid economic expansion have led to more funding for research, thus increasing scientific productivity (42). Then, developed countries tend to have higher animal food consumption and higher obesity rates, and it has been reported that diets containing high volumes of animal fat and protein have a high risk of SBA, with correlation coefficients of 0.61 and 0.75, respectively (43). What’s more, because developed countries have advanced medical level and rich scientific research resources, cooperation with developed countries can promote the improvement of scientific productivity (44). The results suggest that in areas where science is weak, internal collaboration will be more effective than international collaboration (45). Therefore, developed nations are encouraged to broaden their collaborative efforts with more countries and regions, as well as to deepen partnerships with less scientifically advanced areas and developing nations. This aims to enhance global medical treatment standards for SBA.

### 4.3 Key Journals, influential authors, and collaborative networks

*Journal of Clinical Oncology* was the journal with the most published articles, and *Cancer* was the journal with the most cited articles (**Table 3**; **Figure 2**). The impact factor of *Journal of Clinical Oncology* is above 45, the impact factor of *Cancer* is above 5, indicating that the quality of research papers related to SBA is high and has certain academic value. Munkholm’s paper titled *Review article: the incidence and prevalence of colorectal cancer in inflammatory bowel disease* in 2003 has the most citations (**Table 2**). The main contribution of this article (46) is to describe the incidence and prevalence of colorectal cancer in IBD patients and explore the risk factors for colorectal cancer associated with IBD. In the early days, due to the similarity of the site and symptoms and the rarity of SBA, all the treatments, pathogenesis and diagnosis were referred to colorectal cancer.

The most representative and influential authors in the field of SBA research have formed their own core group of authors, and there is mutual cooperation among them (**Figure 3**). Among them, Aparicio T worked most closely with other authors or teams whose team published SBA: French intergroup clinical practice guidelines for diagnosis, treatments and follow-up (SNFGE, FFCD, GERCOR, UNICANCER, SFCD, SFED, SFRO) provide information on the diagnosis and treatment of SBA (47). Rp Dematteo’s team found that Cancer-associated fibroblast secretion of PDGFC promotes gastrointestinal stromal tumor growth and growth metastasis (48).

### 4.4 Historical three stages of changes in the research field of SBA

The research field of SBA is divided into three stages over time (**Figure 6**; **Table 7**), which can better determine the changing trend of research focus and research direction in different periods (49).

#### 4.4.1 Focus in the early stages of research(1992-2007): pathogenesis and treatment of intestinal tumors and related diseases

Crohn et al. first described the characteristics and treatment of regional enteritis in 1932 (50). Neil A. Abrahams et al. emphasized the importance of histopathological parameters as prognostic indicators by analyzing 37 cases of SBA (51). Jesper Ld, WY et al. have shown that the change of intestinal exposure of bile may be the potential biological mechanism (52). M H. Wallace et al. were the first to show that all patients with familial adenomatous polyposis (FAP) develop duodenal adenomas in the early stages, and about 5% of them develop cancer (53). JMD Wheeler et al. studied the genetic pathway of SBA and pointed out that SBA may have a different genetic pathway from colorectal cancer (54). Irmgard E Kronberger et al’s literature review showed that 5-Aminosalicylates are thought to prevent the development of colorectal cancer and small bowel adenocarcinoma in inflammatory bowel disease compared to azathioprine (55).

#### 4.4.2 Focus in the second stages of research(2007-2015): chemotherapy drug development and treatment optimization

Trikudanathan, G. et al. review states that 5-fluorouracil (5-FU) is the mainstay of most advanced/metastatic disease regimens and that the addition of various platinum compounds to 5-FU/capecitabine improves clinical outcomes (56). Xiang, X. J. et al. evaluated the efficacy and safety of oxaplatin in combination with 5-fluorouracil and leucovorin (modified FOLFOX regimen) as first-line chemotherapy, and the results showed that modified FOLFOX regimen showed high efficacy and good tolerability in patients (57).

#### 4.4.3 Focus in the third stages of research(2015-2023): improvements in diagnostic techniques and prognostic factors

Pat Gulhati et al. investigated the effect of bevacizumab in combination with capecitabine and oxaliplatin in the treatment of phase II SBA or ampulla of Vater, showing that the treatment was well tolerated (58). Studies by Thomas Aparicio et al. have shown that gastrointestinal endoscopy can be used as a means of initial diagnosis of SBA (3). The CNN optical pathology diagnostic model trained by Zachariah et al. identified adenomas or hyperplastic polyps with an accuracy of 94% (59). Chen, V. et al’s systematic review suggests that cytoeducational surgery with hyperthermic intraperitoneal chemotherapy (CRS-HIPEC) may be a safe and feasible treatment for patients with SBA with peritoneal metastasis (60). Su Bum Park et al. found that double-balloon enteroscopy is an effective tool for diagnosing small bowel tumors and determining their benign or malignant nature in a retrospective analysis (61). Analysis by Li et al., similar to the topic of this study, also found an outbreak of citations to SBA case reports from 2020 to 2022 (40), suggesting that individualized diagnosis and management of SBA is a trend that has developed over the past two years.

### 4.5 Current hotspots and emerging trends in SBA research

Combining the keyword frequency and keyword clustering analysis, the research hotspots and frontiers are as follows (**Table 6**; **Figure 5**):

#### 4.5.1 #0 chemotherapy: chemotherapy drugs and treatment regimens of SBA

SBA is a clinically and anatomically distinct cancer that lacks prospective data to support optimal management (62). Fluorouracil, leucovorin, and cyclophosphamide (FOLFIRI) regimen including chemotherapy drugs such as 5-FU and its precursors (fluorouracil), leucovorin, and cyclophosphamide is usually used for the treatment of advanced small bowel adenocarcinoma (10). Chemotherapy regimens are usually selected according to tumor stage, overall patient status, and treatment objectives, but none of these regimens has been clearly demonstrated to be superior to others in patients with small bowel adenocarcinoma (29). Therefore, chemotherapy regimens for SBA still require more research to determine the optimal treatment strategy. The study by Li (40) et al., which is similar to our study, also notes that France and the United States have also issued treatment guidelines for small bowel adenocarcinoma (29), a major milestone in the management of SBA patients.

#### 4.5.2 #3 tumor, #4 small intestine, #6 malignant tumor, #8 small bowel carcinoma, #1 inflammatory bowel disease, #2 celiac disease, #5 lynch syndrome: the related diseases of SBA

Compared to colorectal cancer, SBA is more often found in late diagnosis (63). There is ample evidence that patients with celiac disease (64), Lynch syndrome (65), inflammatory bowel disease (66), Crohn’s disease (regional enteritis) (67), and Peutz-Jeghers syndrome (68) have a several-fold higher risk of SBA than the general population. Most of the studies so far have given explanations that patients with these diseases or symptoms (69), the DNA repair mechanisms of intestinal mucosal cells may be impaired, which increases the risk of cell mutations and further increases the incidence of small bowel adenocarcinoma. By elucidating the underlying mechanisms and identifying high-risk individuals, researchers aim to improve early detection, develop targeted therapies, and ultimately reduce the burden of SBA in these vulnerable populations.

#### 4.5.3 #7 disease-specific survival: the prognostic survival time of SBA

According to statistics from the National Cancer Institute in the United States, the 5-year relative survival rate for small intestine cancer was 69.5% from 2013 to 2019, ranging from 84.2% for localized disease to 42.4% for advanced disease (5). Zhang et al. found that patients with certain molecular alterations, such as mutations in the TP53 gene or Ki-67 protein overexpression, were associated with poor disease-specific survival (70). Smith et al. found that patients who received multimodality therapy (including surgery, chemotherapy, and radiation) had significantly improved disease-specific survival compared to patients who received only a single modality (71). These findings highlight the need for a comprehensive approach to treatment of SBA patients to optimize their survival outcomes.

## 5 Conclusions

The number of SBA articles is generally on the rise. Developed countries have more research achievements and closer cooperation among countries and lack cooperation with other countries. The research hotspots focus on chemotherapy, related diseases of SBA, and prognostic survival time. Continued international cooperation and focus on diagnostic techniques and prognostic factors play a vital role in advancing global medical treatment standards for SBA.

## 6 Strength and limitations

To the best of our knowledge, little research systematically and comprehensively discusses the research progress and changing trends in the SBA field. Therefore, to eliminate this limitation, we used CiteSpace software to visualize information such as authors, journals, and keywords. This study is the first to analyze the literature in the field of SBA in the past 100 years. By retrieving the WOSCC database with a large amount of data in the field of SBA, almost all the original studies in this field were included, and the history, current situation and trend analysis of the field of SBA were conducted. On the one hand, this research provides valuable information for scholars in this field, which helps them understand the development process of SBA and master the hot topics at the forefront; on the other hand, it also provides new research perspectives and ideas for exploring the development direction of SBA.

However, this research inevitably has limitations that need to be solved in the future. Due to the continuous updating of the database and the limited analysis year span of CiteSpace software, only the articles from 1923 to December 31, 2023, were selected for this research, and articles published after that were not included in this research. Therefore, there will be discrepancies between bibliometric analysis and actual publication. Restricted by the capabilities of the analysis software, only articles in the WOSCC database are included in this research, which may result in potentially incomplete analytical data. Due to the limitations of CiteSpace software, a lack of unified parameter setting standards, data loss and partial data overlap will inevitably occur in the process of software clustering, which will also lead to the deviation of analysis results. In addition, according to the general process of bibliometrics research, this study adopts an accurate literature search strategy to ensure the correlation between downloaded literature and research topics and uses CiteSpace software to remove duplicate literature. However, it is still possible to have subtle errors due to the database or software issues, which are slightly insufficient in artificial induction.
